# Scrapie prevalence in sheep of susceptible genotype is declining in a population subject to breeding for resistance

**DOI:** 10.1186/1746-6148-6-25

**Published:** 2010-05-14

**Authors:** Thomas J Hagenaars, Marielle B Melchior, Alex Bossers, Aart Davidse, Bas Engel, Fred G van Zijderveld

**Affiliations:** 1 Central Veterinary Institute of Wageningen UR, P.O. Box 65, 8200 AB Lelystad, the Netherlands; 2 Biometris, Wageningen University and Research Centre, P.O. Box 100, 6700 AC Wageningen, the Netherlands

## Abstract

**Background:**

Susceptibility of sheep to scrapie infection is known to be modulated by the PrP genotype of the animal. In the Netherlands an ambitious scrapie control programme was started in 1998, based on genetic selection of animals for breeding. From 2002 onwards EU regulations required intensive active scrapie surveillance as well as certain control measures in affected flocks.

Here we analyze the data on genotype frequencies and scrapie prevalence in the Dutch sheep population obtained from both surveillance and affected flocks, to identify temporal trends. We also estimate the genotype-specific relative risks to become a detected scrapie case.

**Results:**

We find that the breeding programme has produced a steady increase in the level of genetic scrapie resistance in the Dutch sheep population. We also find that a significant decline in the prevalence of scrapie in tested animals has occurred a number of years after the start of the breeding programme. Most importantly, the estimated scrapie prevalence level per head of susceptible genotype is also declining significantly, indicating that selective breeding causes a population effect.

**Conclusions:**

The Dutch scrapie control programme has produced a steady rise in genetic resistance levels in recent years. A recent decline in the scrapie prevalence per tested sheep of susceptible prion protein genotype indicates that selective breeding causes the desired population effect.

## Background

Scrapie in sheep is a transmissible spongiform encephalopathy (TSE) present in most sheep-producing countries [1,2]. Infection with (classical) scrapie is thought to occur at young age, after which it takes an incubation period of one or more years before clinical signs, such as uncoordinated movement, abnormal postures and severe scratching, become apparent. During this incubation period the infectious prion protein PrP^Sc ^accumulates in the animal [3]. Scrapie control has become a priority in many countries mainly because experimental infection of sheep with bovine spongiform encephalopathy (BSE) has shown that sheep can be infected via the oral route and that the resulting clinical symptoms are very similar to scrapie [4]. Fears that BSE may have been introduced into sheep through consumption of feed supplements in the past, with potential consequences to public health [5,6], have eased somewhat recently since tests of millions of sheep have not produced a single sample positive for BSE, neither in the healthy-slaughter nor in the fallen-stock testing stream.

The susceptibility to scrapie is modulated by polymorphisms of the sheep prion protein (PrP) gene. In this paper we focus on classical scrapie, for which the most important polymorphisms occur at the codons 136, 154 and 171. Five alleles (VRQ, ARQ, AHQ, ARH and ARR) are observed in this study. The VRQ allele is known to confer high susceptibility to classical scrapie, the ARQ and ARH alleles are associated with moderate susceptibility and the AHQ allele with low susceptibility. The ARR allele confers resistance, with the homozygous genotype ARR/ARR being extremely resistant [1,7]. These properties make the use of exclusively ARR/ARR rams for breeding (to be referred to as *ram selection *below) a means to reduce the number of susceptible animals in a sheep population.

In the European Union (EU) the Regulation EU 2001/999 prescribes the genetic testing, and the selection of rams intended for breeding in scrapie-free flocks of "high genetic merit" (followed by culling of the rams with a VRQ allele). Several years before this EU regulation came into force, some Member states already had a national breeding programme in place, including The Netherlands (started in 1998), Great Britain (started in 2001) [8-12], and France (started in 2002). In the Netherlands, the breeding programme consisted of ARR/ARR ram selection, and initially sheep breeders could join it on a voluntary basis. This programme was made compulsory for the whole Dutch sheep industry in November 2004, thereby becoming the most ambitious programme worldwide.

Another important activity for the control of scrapie is the large active surveillance programme of testing healthy slaughtered sheep and fallen stock for scrapie by a rapid test on brainstem samples. This programme concerns animals over 18 months of age and was introduced in the EU in 2002 [13]. The number of animals to be tested is prescribed yearly by the EU and is a percentage of the total size of the Member states' slaughtered sheep and fallen stock. In the Netherlands in the period 2002-2008 this percentage ranged between 10 and 25% for healthy slaughtered sheep and 2.7% and 11% for fallen stock. Since 2003, the EU Regulation EU 2001/999 governs the control measures in flocks of origin of classical-scrapie positive animals in the active or passive surveillance. These measures consist of either a whole-flock cull or genotyping all animals and culling the animals of susceptible genotype and examining the brain stem of all or a sample of the culled animals of at least 12 months of age for scrapie positivity, using rapid tests.

The Dutch sheep population consists of a breeding sector with three large pure breeds (Texel, Swifter and Zwartbles), more than 30 smaller breeds, and a production sector dominated by Texel and Swifter sheep. The production sector makes up more than 90% of the Dutch sheep population. The Dutch Agricultural Census counted 14.369 farms with sheep in The Netherlands in 2005, 7286 of which had less than 50 sheep (Statistics Netherlands, Agricultural census database: http://www.cbs.nl). However, as only farms with certain minimum economic value of their overall agricultural activities are included in the census, the total number of farms with sheep, including those that keep sheep for recreational purposes, is much higher [14].

In Table 1 we give a chronological overview of the scrapie control measures in The Netherlands since the disease became notifiable in 1993. Ram selection was obligatory between October 2004 and September 2005 for all sheep farmers with more than 10 breeding ewes (certain small breeds being exempted), and between September 2005 and June 2007 for all sheep farmers (including those with less than 10 breeding ewes). In June 2007 the legal obligation was lifted. This compulsory ram selection programme had been preceded by a voluntary program for sheep breeders that started in 1998. In addition to the scrapie control measures listed in Table 1, the BSE-related EU ban on use of MBM and most other animal protein in feed of farm animals, imposed in 2000 [15] could potentially have impacted on scrapie trends: if sheep feed cross-contaminated with scrapie would have been a significant scrapie transmission route before 2000 [16], one would expect the ban to lead to a significant reduction in scrapie prevalence becoming noticeable around 2003 (taking into account a few years delay due to incubation time).

**Table 1:** Scrapie control measures

Year/date of introduction	Control measure
1993	Scrapie becomes a notifiable disease
2002 January	Active surveillance (EU)
2003 October	Control measures in flocks of origin of classical-scrapie positive animals (EU)
2004 October	Obligatory use of ARR/ARR rams for flocks with more than 10 breeding ewes (except some rare breeds)
2005 September	Obligatory use of ARR/ARR rams for all flocks (except some rare breeds)
2007 June	Obligatory use of ARR/ARR rams withdrawn

Chronology of scrapie control measures in The Netherlands.

The three main aims of this paper are to identify trends in scrapie prevalence in slaughtered and fallen sheep in The Netherlands, to identify trends in the frequencies of different genotypes in the Dutch sheep population, and to determine the relative risk of different PrP genotypes to become a detected scrapie case.

## Methods

### Data and analyses

The analysis of the abovementioned trends and risks are carried out based on recently gathered data on both scrapie infection of and genotype frequencies in the Dutch sheep population. These data consist of two parts: surveillance data and culled-flocks data. The surveillance data consist of the scrapie test results accumulated within the Dutch active surveillance on TSEs in sheep (2002-2008), and of a yearly random genotyping sample from this active surveillance (2005-2008), both from the healthy-slaughter and the fallen-stock samples. Details on the sampling strategy, genotyping technique and rapid test used are given below. The culled-flocks data (2003-2008) consist of scrapie genotyping results and scrapie infection test results in animals that were culled, as part of the mandatory scrapie control efforts, on flocks of origin of scrapie index cases. For details on genotyping and testing see below. Immunohistochemistry (IHC) was used for confirmation of the positive cases detected using the rapid test. IHC and Western blotting were used to discriminate between classical and atypical scrapie.

The surveillance data allow us to study any temporal trend in the detected scrapie cases in the Dutch sheep population and at the same time provide a sample from the genotype frequencies at a national level. The combination allows us to study any related trends in case numbers and frequencies of susceptible versus resistant genotypes. Clearly, we expect to see an increase in the frequency of resistant genotypes, and as a result of that, after some delay, a reduction in scrapie case numbers.

When assessing prevalence trends, we are interested in particular to see if not only the overall prevalence but also the prevalence calculated *per head of susceptible genotype *is declining as a result of the breeding programme. This is because we seek to determine if the breeding programme causes a population effect. The expected population effect is derived using a simple mathematical model in the additional material [Additional file 1].

Apart from the ram selection, also the culling of scrapie flocks will impact on the scrapie transmission potential, measured by the basic reproduction number R_0_, in two ways. Firstly, in comparison to a situation without culling, the culling of flocks of origin of scrapie cases will reduce the mean duration of flock-level scrapie outbreaks, and thereby reduce the length of the period that infected flocks pose an infection risk to other (still unaffected) flocks. For a mathematical model of this latter effect we refer the reader to the additional material [Additional file 1]. A second way in which the culling of affected flocks impacts on the transmission potential is by reducing the frequency of susceptible genotypes in the population [9,11,12], thus aiding the ram selection program in increasing the genetic resistance level.

The culled-flocks data allow us to calculate the detected infection prevalence in different genotypes, and thereby obtain information on the relative risk of infection across different genotypes.

### Genotyping

PrP genotypes were determined (at codons 136, 154, and 171) by a routine TaqMan test that is completely automated. It can detect polymorphisms 136 A to V, 154 R to H, and 171 Q to R. From 2005 (genotyping sample from surveillance) or 2006 (genotyping of index cases in the surveillance and of culled animals in flocks of origin) onwards our TaqMan genotyping additionally distinguishes between Q and H at codon 171. When analyzing the culled-flocks data we consider total numbers of animals for each genotype across the period 2003-2008 and we therefore group the 2006-2008 ARQ and ARH results together, using the notation ARQ*. The TaqMan principle is a test in which a small part of the PrP gene is amplified. During amplification dedicated fluorescent probes are used to detect absence/presence of specific polymorphisms. A second test, based on pyrosequencing, was used as a confirmatory test on randomly selected samples.

### Sampling and testing in the active surveillance

Throughout the period of 2002-2008 the sampling strategy in the Dutch surveillance programme at each of the slaughterhouses (healthy slaughter) consisted of randomly selecting one animal per *n *slaughtered sheep, with a minimum of one sheep on a slaughter day per slaughterhouse. Here *n *was chosen such that the expected total number of animals sampled in The Netherlands on a 12-months basis would match EU requirements. Due to changes in the EU target number of animals tested per 12 months (from 1 January 2002 until 22 August 2002: 14,250; from 22 August 2002 until 1 January 2004: 39, 500; from 1 January 2004 until 8 July 2006: 10,000; from 8 July 2006 until 1 July 2007: 23,300 and from 1 July 2007 onwards: 10,000), *n *changed between periods, with *n *ranging between 3 and 10.

The sampling of fallen stock at the rendering plant (i.e., at the sole such plant in The Netherlands) also followed a random strategy throughout the period of study. During the period 2002-2008, the sampling strategy was designed to randomly sample sufficient fallen sheep to fulfill the EU requirements of testing a specified minimum number of fallen sheep per 12 months. Most often the minimum number was 10,000 animals per 12 months, and this target could be achieved by randomly sampling at most 72 sheep per working day at the rendering plant. In other periods the sampling strategy was randomly adjusted to the changes in the EU target number of fallen sheep tested per 12 months (from 1 January 2002 until 22 August 2002: 3,000; from 22 August 2002 until 1 January 2004: 5,000; from 1 January 2004 until 8 July 2006: 10,000; from 8 July 2006 until 1 July 2007: 20,000 and from 1 July 2007 onwards: 10,000). The rapid tests used for both the active surveillance and culled flocks were the Prionics Check Western (2002-2006) and the Prionics Check Western SR from June 2006 onwards. For classical scrapie these tests have the same diagnostic and analytical sensitivity in our hands. As a result, we do not expect any temporal bias in the data from the Dutch surveillance programme in the period of study.

### Sampling from the active surveillance

From the brain homogenates from healthy slaughter arriving for testing at the Central Veterinary Institute, approximately 1 out of 13 (2005, 2007) or 23 (2008) or 33 (2006) was randomly selected for genotyping. From the brain homogenates from fallen stock approximately 1 out of 23 (2005, 2007 and 2008) or 44 (2006) was randomly selected for genotyping. In Table 2 we give the total numbers of samples taken from healthy slaughtered sheep and from fallen stock, together with the size of the random sample for genotyping. Due to the random sampling at the slaughterhouse and at the rendering plant, we expect each yearly random sample to be representative for the allele and genotype frequencies in animals over 18 months of age in that year in healthy slaughtered sheep and in fallen stock, respectively.

**Table 2:** Scrapie surveillance

Year	2002	2003	2004	2005	2006	2007	2008
**Active surveillance**							
Healthy slaughter	19642	21140	8949	8910	18564	15813	10214
Fallen stock	3864	4000	10137	10085	17528	14990	10193
Total	23506	25140	19086	18995	36092	30803	20407

**Size of sample genotyped**							
Healthy slaughter	N/A	N/A	N/A	663	551	1222	446
Fallen stock	N/A	N/A	N/A	433	397	676	446
Total	N/A	N/A	N/A	1096	948	1898	892

Number of samples from the scrapie surveillance, and number of samples selected randomly for genotyping.

### Relative scrapie risks

We will use the genotype ARQ*/VRQ, being the most frequent genotype amongst Dutch scrapie cases, as the reference for defining genotype-specific relative risks. The mathematical definition and statistical estimation of genotype-specific relative scrapie risks are as follows. If the scrapie risk of ARQ*/VRQ animals is given by a (binomial) probability *p*_ARQ*/VRQ _of being found positive, we write the corresponding probability of genotype *γ *as *p*_*γ *_= *r*_*γ *_*p*_ARQ*/VRQ_, where *r*_*γ *_is the relative risk of genotype *γ*. The parameters *r*_*γ *_are estimated using maximum-likelihood based on binomial probabilities *p*_*γ *_and confidence bounds are based on the likelihood ratio test.

## Results and Discussion

### Trends in genotype frequencies

**Figure 1:**
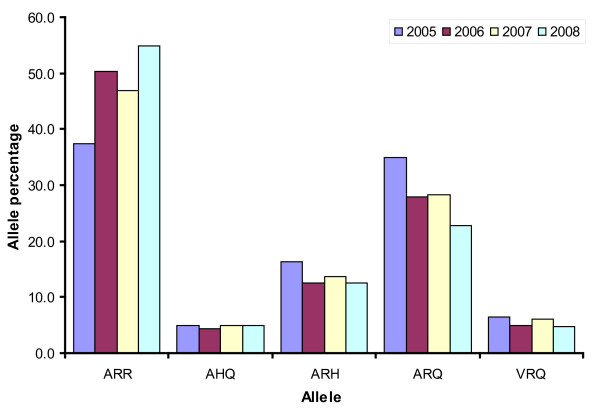
**Trends in allele frequencies**. Allele frequencies found in yearly samples from the active surveillance.

**Figure 2:**
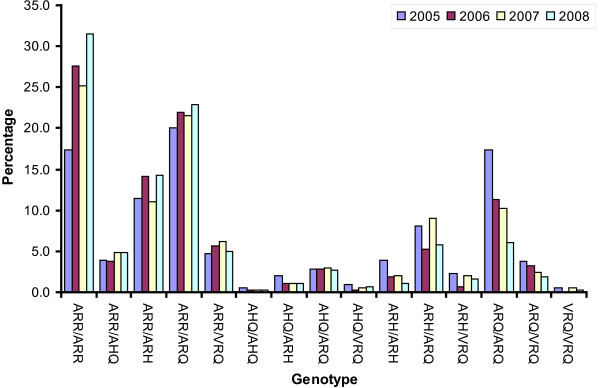
**Trends in genotype frequencies**. Genotype frequencies found in yearly samples from the active surveillance.

Figures 1 and 2 display the trends in frequencies of the alleles and genotypes, respectively, in the yearly genotyping sample from the active surveillance (2005-2008). In Figure 1 we observe that the frequency of the ARR allele has increased from less than 40% in 2005 to more than 50% in 2008, which is a significant increase (p < 0.0001, chi-square test). The observed decline in 2007 in comparison to 2006 is not significant (p = 0.086). We therefore conclude that ram selection has produced a significant rise in the frequency of the ARR allele in the Dutch sheep population. We also observe a reduction in the presence of the ARQ allele. No significant trends are observed for the ARH, AHQ and VRQ alleles. In terms of genotypes (Figure 2) the trend towards more scrapie resistance is best visible in the genotypes ARR/ARR, ARR/AHQ and ARR/ARQ (overall increase) and ARH/ARH, ARQ/ARQ and VRQ/ARQ (overall decrease). For the less frequent genotypes the patterns in Figure 2 are dominated by chance fluctuations. The total frequency of animals without ARR allele calculated from the 2005 sample is 42.5%, and in 2008 this number is 21.5%. The overall downward trend suggests an average yearly reduction of this frequency with 20.2%.

### Trends in scrapie prevalence

**Figure 3:**
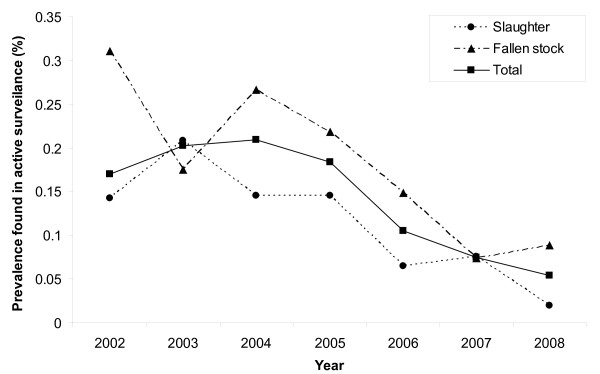
**Trend in scrapie prevalence**. The temporal trend of classical scrapie prevalence in the active scrapie surveillance in the period 2002-2008.

Table 3 provides an overview of all scrapie cases found in the different surveillance streams in The Netherlands in the period 2002-2008. In this period there have been only four cases of atypical scrapie; we therefore restrict the analyses to classical scrapie. By virtue of the large number of sheep tested, the active surveillance stream provides the best insight in the temporal trend in the underlying scrapie infection prevalence in recent years. In contrast, the number of cases amongst clinically suspect animals are too low to allow conclusions about the trend. Prevalence of scrapie detected in the active surveillance is generally higher in fallen stock than in normal slaughter (Chi-square test on the totals for the period 2002-2008 yields P = 0.023). The prevalence in the active surveillance, shown in Figure 3, is significantly (chi-square test, P = 0.0002) declining from close to 2 cases per 1000 tested animals in 2004 to approximately 0.5 in 2008. We note that the decline is occurring too late in time to be caused by the EU ban on MBM in animals feed introduced in 2000. In Table 4 we also list the numbers of flocks that have been (partially) culled, being traced as flocks of origin of index cases from the surveillance, and the further cases detected in culled animals from these flocks.

**Table 3:** Scrapie cases

Year	2002	2003	2004	2005	2006	2007	2008
**Active surveillance, ** **classical scrapie**	Number of positive cases *Percentage of tested animals * *(confidence bounds)*						

Healthy slaughter	28 *0.15* *(0.10-0.21)*	44 *0.21 * *(0.15-0.28)*	13 *0.16 * *(0.08-0.25)*	13 *0.16 * *(0.08-0.25)*	12 *0.07 * *(0.04-0.11)*	12 *0.08 * *(0.04-0.13)*	2 *0.03 * *(0.00-0.08)*
Fallen stock	12 *0.34 * *(0.17-0.54)*	7 *0.20 * *(0.08-0.37)*	27 *0.28 * *(0.18-0.39)*	22 *0.23 * *(0.14-0.33)*	26 *0.15 * *(0.10-0.22)*	11 *0.08 * *(0.04-0.13)*	9 *0.10 * *(0.04-0.17)*
Total	40 *0.17 * *(0.12-0.23)*	51 *0.21 * *(0.15-0.27)*	40 *0.21 * *(0.15-0.29)*	35 *0.19 * *(0.13-0.26)*	38 *0.11 * *(0.08-0.14)*	23 *0.08 * *(0.05-0.11)*	11 *0.06 * *(0.03-0.10)*

**Active surveillance, ** **atypical scrapie**	0	0	0	2	0	2	0

**Clinical suspects **Cases (Number of suspects)	6 (13)	0 (0)	0 (2)	0 (2)	5 (9)	0 (0)	1 (3)

Yearly number of scrapie cases in The Netherlands over the period 2003-2008, in various surveillance streams.

**Table 4:** Scrapie flock culls

Flock culls in 2003-2008	classical	atypical	all
Number of flock culls in 2003-2008	69	2	71
Animals present at the time of flock cull	18224	128	18352
Animals culled (including later individual culls)	6507	60	6567
Animals tested	4304	60	4364
Number of secondary cases (i.e. in culled flocks)	191	0	191
Number of culled flocks with secondary cases	49	0	49

Summary information of scrapie flock culls in 2003-2008.

**Figure 4:**
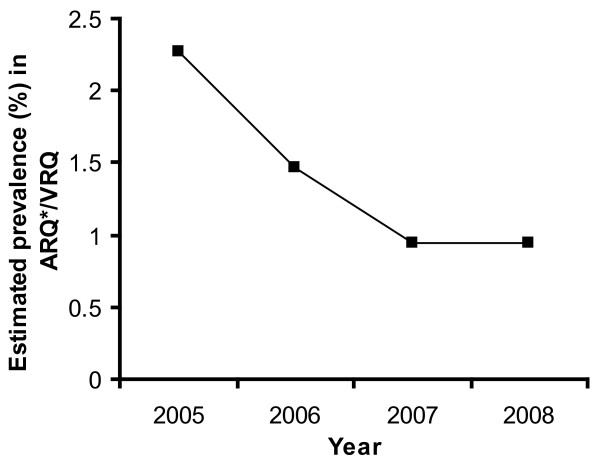
Trend in prevalence of scrapie in ARQ*/VRQ. Trend in the estimated prevalence of classical scrapie in animals of ARQ*/VRQ genotype in the active surveillance in the period 2005-2008.

Is the decline in scrapie prevalence as found in Figure 3 simply a direct result of the reduction (by the ram selection programme) in the frequency of susceptible genotypes, or has the programme also led to a population effect? To answer this question, we need to inspect the prevalence per tested animal of susceptible genotype. In Figure 4 we do this for the years 2005-2008, focusing on the genotype ARQ*/VRQ, the most prominent genotype amongst scrapie positive animals (68 out of 107 cases). Here we have used a result from the additional material [Additional file 1: Equation (A.2)] to estimate the prevalence in ARQ*/VRQ animals tested in each year from the overall prevalence in tested animals, the proportion of positive animals having ARQ*/VRQ genotype, and the proportion of animals having ARQ*/VRQ genotype within the sample from the active surveillance of that year. The likelihood-ratio test shows that there is a significant (conventional chi-square approximation, P = 0.009) downward trend in prevalence from 2005 to 2008 [Additional file 1]. As the sampling strategy remained the same throughout this period, and the test sensitivity is believed to have remained the same as well, we conclude from the observed decline that a reduction in the scrapie infection risks to animals of susceptible genotype has occurred. We interpret this reduction as being a population effect caused mainly by the increase in the number of animals of resistant genotype (for details we refer to the additional material [Additional file 1]). As we argue in the Conclusions, the effect on transmission of the reduction of the infectious period of affected farms (by their detection and culling) is expected to be less important.

### Flock culls

In Table 4 and 5 we give summary information on the scrapie flock culls in 2002-2008. In this period, in total 71 flock culls were carried out, the yearly number peaking at 18 flock culls in 2005. Two flock culls were related to an atypical scrapie index case, and these produced no secondary cases. Of all animals present in the flocks at the time of the flock cull, close to 36% was culled, and of these culled animals approximately 66% was tested, producing 191 secondary cases of classical scrapie. We note that the number of animals actually infected at the time of culling is expected to be higher than that, as infected animals will be tested positive only when they are at a sufficiently advanced stage of incubation [17,18]. Mathematical modelling would be needed to derive infection prevalence estimates from detectable prevalence; for examples of such modelling applied to clinical case surveillance data in Great Britain, see [19,20].

**Table 5:** Scrapie flock culls by year

Flock culls in 2003-2008	2003	2004	2005	2006	2007	2008	Total
Number of flock culls (all)	3	16	18	16	9	9	71
Number of flock culls (classical)	3	16	17	16	8	9	69
Number of secondary cases	5	79	30	46	21	10	191

Summary information of scrapie flock culls in 2003-2008 by year.

### Genotype profiles of scrapie-affected flocks

In Tables 6 and 7 we list the genotype and allele frequencies across culled flocks with classical scrapie in the period 2002-2008. Before making a comparison between these frequencies and those across the whole Dutch population as found in the yearly genotyping sample from the active surveillance in the period 2005-2008, we note that the result of such a comparison should be interpreted with care. This is because the frequencies in the culled flocks data represent the genetic variation across animals present in the flock (and aged at least 12 months), whereas the yearly genotyping samples from the active surveillance represents the genetic variation in slaughtered animals and fallen stock (and above 18 months of age). In the presence of an ongoing ARR/ARR ram selection policy, younger animals are expected to have a higher ARR frequency than older animals, and therefore the expected higher age of the animals tested in the active surveillance will tend to produce a lower observed ARR allele frequency than if animals were sampled as in the culled-flocks data. We find that, at least at the moment of culling, the mean allele and genotype frequencies in the culled scrapie flocks are very similar to what is observed in the active surveillance. For example, the mean ARR frequency across flocks culled in the period 2005-2008 is 51.5%, and compares to a range [37.5% (2005), 54.9% (2008)] spanned by the yearly genotyping samples from the active surveillance. Similarly, the mean ARQ* frequency across flocks culled in the period 2005-2008 is 38.0%, comparing to a range [35.3% (2008), 51.2% (2005)] spanned by the yearly genotyping samples from the active surveillance.

**Table 6:** Culled-flocks genotype frequencies

Genotype	Number	Percentage
ARR/ARR	4405	24.2
ARR/AHQ	502	2.8
ARR/ARQ*	6810	37.4
ARR/VRQ	1290	7.1
AHQ/AHQ	59	0.3
AHQ/ARQ*	334	1.8
AHQ/VRQ	48	0.3
ARQ*/ARQ*	3775	20.7
ARQ*/VRQ	860	4.7
VRQ/VRQ	78	0.4
Untyped	63	0.3

Total	18224	100

Genotype distribution in culled flocks with classical scrapie in 2002-2008.

**Table 7:** Culled-flocks allele frequencies

Allele	Number	Percentage
ARR	17412	47.9
AHQ	1002	2.8
ARQ*	15554	42.8
VRQ	2354	6.5

Total	36322	100.0

Allele distribution in culled flocks with classical scrapie in 2002-2008.

### Relative susceptibilities of different genotypes

Table 8 shows the scrapie cases found in culled flocks, and the risk, in these flocks, to different genotypes of being found test positive. Results are shown based on all culled flocks; when calculated based on culled flocks with secondary cases, the risk *r*_*γ *_relative to the genotype ARQ*/VRQ is not significantly different (data not shown).

**Table 8:** Relative scrapie risks

Culled flocks				British cases (1998-2002)
**Genotype**	**Cases ** **(Total tested)**	**Cases/1000**	**Relative risk ** **(Confidence ** **bounds)**	**Relative risk ** **calculated from ** **Ref. **[7]

ARR/ARR	0 (237)	0.0	0.0 (0.0 -0.03)	0.00
ARR/AHQ	0 (26)	0.0	0.0 (0.0 -0.26)	0.00
ARR/ARQ*	0 (409)	0.0	0.0 (0.0 -0.02)	0.00
ARR/VRQ	24 (875)	27.4	0.10 (0.06-0.15)	0.03
AHQ/AHQ	0 (32)	0.0	0.0 (0.0 -0.21)	0.02
AHQ/ARQ*	1 (192)	5.2	0.02 (0.001-0.08)	0.04
AHQ/VRQ	2 (26)	76.9	0.27 (0.05-0.79)	0.00
ARQ*/ARQ*	54 (1882)	28.7	0.10 (0.08-0.14)	0.13
ARQ*/VRQ	155 (553)	280.3	1.00 (1.00-1.00)	1.00
VRQ/VRQ	21 (57)	368.4	1.31 (0.88-1.83)	2.33

Cases found in different genotypes (including index case) in culled flocks, cases per 1000 animals of given genotype, and relative case risk *r*_*γ *_of the different genotypes (setting the risk of ARQ*/VRQ equal to unity). Comparison to the relative risks of death from scrapie in British sheep estimated by Baylis et al. [7]. The total number of cases in this Table is 257; three remaining cases were in animals for which the genotype could not be determined.

In Table 8 we compare our results with relative risks of clinical scrapie in British sheep calculated from risk estimates by Baylis et al. [7] from British passive scrapie surveillance data from the period 1998-2002. The same surveillance data have been analyzed by Tongue et al. [21], but with different genotyping dataset(s) as denominator data. These authors analyzed the risks of clinical scrapie relative to the genotype ARQ/ARQ by estimating odds ratios for the different genotypes. The (point) estimates they obtain, when recalculated relative to ARQ/VRQ, are broadly similar to the relative risks we have calculated here from the estimates by Baylis et al. One striking difference between the two sets of relative risks in Table 8 is observed for the ARR/VRQ genotype: for this genotype the relative infection risk estimated here from the Dutch culled-flocks data is higher than the risk of death from scrapie as estimated by Baylis et al. One possible hypothesis would be that the difference arises due to breed [22] and/or scrapie strain differences between the countries. Another possible hypothesis would be that the difference arises because ARR/VRQ animals affected by scrapie are less likely to show overt clinical symptoms, and thus to be detected by the British passive surveillance system (1998-2002), than animals of other susceptible genotypes.

## Conclusions

In this paper we have analyzed scrapie prevalence data obtained from both surveillance and control, together with yearly genotyping sample from the active surveillance. Although these analyses are specific to The Netherlands, the results seem relevant and encouraging for all other countries interested in scrapie control.

The main results are as follows. Scrapie prevalence in The Netherlands is showing a downward trend in the last four years. Allele and genotype distributions in the Dutch sheep population are showing a clear trend of increasing genetic resistance to scrapie, showing that compliance to the ram selection programme has been substantial. Estimated prevalence levels per head of susceptible genotype are declining significantly, consistently with an anticipated population effect of the breeding programme. Finally, we observe that the relative risk found here for ARR/VRQ animals is much larger than their relative scrapie risk under past passive surveillance in Great Britain.

The observed reduction in scrapie prevalence is likely to be due to two causes: the increasing genetic resistance of the population and culling of scrapie flocks. How much may be attributed to the increase in scrapie resistance, and how much to the shortening of flock-level outbreaks due to the culling of affected flocks? An order-of-magnitude estimation using modelling arguments and using data for 2005 suggests that the contribution of affected-flock culling to the reduction of scrapie prevalence in The Netherlands is small compared to that of selective breeding. For details we refer the reader to the additional material [Additional file 1].

As reported elsewhere [23], a comparison of the genotyping samples from the active surveillance to an independent genotyping sample, taken on 168 sheep farms, shows a good correspondence. More precisely, in [23] it is found that the temporal trend in genotype frequencies in the yearly genotyping sample from the active surveillance conforms well to the trend visible in the sample taken on the farms when consecutively removing recent birth cohorts. This result provides further confidence for the assumption that the yearly genotyping sample from the active surveillance streams provides a representative picture of the genotype distribution in the Dutch sheep population. Comparing the overall genotype frequencies estimated above from the surveillance data and from the genotype profiles across the culled flocks, we can investigate to which extent these latter flocks have more susceptible profiles as compared to the national average. We observe that frequencies of susceptible genotypes do not seem to be much above average on farms with scrapie outbreaks, which may seem paradoxical. However, in the presence of ARR ram selection, current between-flock differences in genotype and allele frequencies might not be representative for the differences at the time that the actual scrapie infections took place. Also, the incomplete tracing of flocks of origin of scrapie cases found in the active surveillance might introduce a bias in the culled flocks data towards more professionally organized sheep farmers, that might be more likely to comply with ram selection. Due to the absence of age information in the culled flocks data it requires genetic model extrapolations to correct for these ram selection effects; such analyses will be reported elsewhere.

The relative scrapie risks of different genotypes in positive flocks provide useful clues to the relative susceptibility of these genotypes. The relative susceptibility is of particular interest as a parameter for mathematical models of within-flock scrapie transmission [24-27]. We note that relative prevalence and relative susceptibility cannot be simply equated to each other for two main reasons. The first is that differences in the rate of disease progress between genotypes (as apparent from incubation time differences) are expected to lead to genotype-dependent probabilities of detecting infection in a scrapie test at a given age. This issue is complicated further by genotype-dependent differences in sensitivities of different rapid tests that have been approved for use in the EU screening program [28]. The second is that infection prevalence will only be proportional to susceptibility away from infection saturation levels, i.e. when prevalence is low.

## Authors' contributions

TH and FGvZ conceived of the study and carried out the analyses. The approach, results and interpretation were discussed between all authors. AB and AD collected the random samples from the surveillance and carried out the PrP genotyping. BE developed the trend analysis of the prevalence in ARQ*/VRQ animals. TH and MBM drafted the manuscript, and the other authors commented on it. All authors read and approved the final manuscript.

## Supplementary Material

Additional file 1 **Details of quantitative analyses**. A text document providing mathematical details supporting the Methods section, providing an order-of-magnitude estimation of the contribution of affected-flock culling to the reduction of scrapie prevalence in the Netherlands, and providing mathematical details of the trend analysis of the scrapie prevalence in ARQ*/VRQ animals in 2005-2008. Click here for file
