# Supplementary material for: Scrapie prevalence in sheep of susceptible genotype is declining in a population subject to breeding for resistance
Source: BMC Vet Res. 2010 May 14;6:25. doi: 10.1186/1746-6148-6-25 (PMC2883980; doi:10.1186/1746-6148-6-25)
Supplement: Additional file 1 — Details of quantitative analyses. A text document providing mathematical details supporting the Methods section, providing an order-of-magnitude estimation of the contribution of affected-flock culling to the reduction of scrapie prevalence in the Netherlands, and providing mathematical details of the trend analysis of the scrapie prevalence in ARQ*/VRQ animals in 2005-2008. [file 1746-6148-6-25-S1.DOC]

**Details of quantitative analyses**

*Additional file to “Scrapie prevalence in sheep of susceptible genotype is declining in a population subject to breeding for resistance” by Thomas J. Hagenaars, Marielle B. Melchior, Alex Bossers, Aart Davidse, Bas Engel, and Fred G. van Zijderveld.*

**1. Population effect**

### This section provides mathematical details supporting the Methods section. In Figure I we provide a schematic context to some of the mathematical quantities introduced below.

Breeding program

Genetic composition of population changes towards more scrapie resistance: increase in *n*_R_

Culling of flocks of origin of cases

Genetic composition of partially culled sheep flock changes towards high scrapie resistance: increase in *n*_R_

Culled sheep flock ceases to present infection risk to other flocks: reduction in *T*_inf_

Reduction of scrapie transmission potential (*R*_0_)

**Figure I.** Overview of the expected effects of the scrapie control measures in Dutch sheep**,** listing three mathematical quantities introduced in this Additional file.

The argument for expecting a population effect of the breeding programme can be made more precise as follows. In a simple SIR (susceptible–infected–removed) model description of scrapie transmission, the endemic equilibrium fraction of infected individuals $i^{*}$is given by $i^{*}=1-\frac{1}{R_{0}}$, where $R_{0}$ is the basic reproduction number, which measures the transmission potential [2]. In the presence of genetically resistant individuals comprising a fraction $n_{R}$ of the population, this equality generalizes to $\frac{i^{*}}{1-n_{R}}=1-\frac{1}{R_{0}\left(0\right)\left(1-n_{R}\right)}$, where $R_{0}\left(0\right)$ is the basic reproduction number for $n_{R}=0$. This result predicts that the equilibrium prevalence per head of susceptible genotype, $\frac{i^{*}}{1-n_{R}}$, will decline when the frequency of resistant genotypes $n_{R}$ is increasing. This same result also predicts a critical (but non-zero) level of remaining susceptibility $1-n_{R}^{c}=\frac{1}{R_{0}\left(0\right)}$, below which scrapie transmission will be insufficient to maintain itself, i.e. scrapie control would be achieved, very much akin to a critical vaccination coverage [1].

**2. Order-of-magnitude estimation of the contribution of affected-flock culling to the reduction of scrapie prevalence in the Netherlands**

*2.1 Contribution of flock culling to control*

The contribution of affected flock culling to scrapie control can be discussed in mathematical terms as follows. If we denote the rate of detection (and subsequent culling) of affected flocks by $\delta,$ and if we write the basic reproduction number $R_{0}$ as $R_{0}=\beta_{f}T_{\inf}$, where$\beta_{f}$ is a transmission parameter and $T_{\inf}$ the mean infectious period of affected flocks, we find

$R_{0}\left(\delta\right)=\beta_{f}\frac{T_{\inf,0}}{1+T_{\inf,0}\delta}$, (A.1)

where $T_{\inf,0}$is the mean of the infectious period in the absence of culling of affected flocks ($\delta=0$). Here the infectious period in the absence of culling of affected flocks has been modelled by an exponential distribution.

*2.2 Order-of-magnitude estimation*

How much of the reduction in scrapie incidence in the Netherlands may be attributed to the increase in scrapie resistance, and how much to the shortening of flock-level outbreaks due to the culling of affected flocks? An “order-of-magnitude” estimation using Equation (A.1) above runs as follows. Here we use the data for 2005. In this year approximately 0.18% of tested animals was found positive for classical scrapie in the active surveillance. In other words, the probability that an animal is found positive in the active surveillance is 0.0018. This probability can be considered as the product of the probability that the animal is from an affected flock, and the prevalence of detectable infections in affected flocks in normal slaughter and fallen stock of at least 18 months of age. As a result of disease incubation, the latter prevalence is expected to be somewhat larger than the overall detectable infection prevalence in culled flocks, which in 2005 was approximately 1.3%. Let us assume it is twice as high, i.e. 2.6%. Then the probability that an animal tested in the active surveillance is from an affected flock is calculated as 0.0018/(0.026)≈0.07. If this probability is a good measure of the prevalence of affected flocks, we thus find that 7% of flocks is estimated as being affected. Assuming that the total number of sheep flocks is 35.000 (based on Ref. 14 in the main text), this corresponds to 2450 affected flocks. With 17 flocks of origin being traced and culled in 2005, the probability per year of culling of an affected flock is $\delta=0.7\%/\text{year.}$ Assuming an average within-flock scrapie outbreak duration of 5 years in absence of affected-flock culling, following Eq. (A.1) the presence of affected-flock culling would reduce the basic reproduction number by about 3.5%. This is small in comparison to a rough estimate of the reduction of the basic reproduction number due to the increase in resistance in the population in 2005, obtained along the lines of Section 1 above as follows. Identifying $n_{R}$ with the population fraction of genotype ARR/ARR (which is a conservative approach), the increase from $n_{R}\left(2005\right)=17.4\%$ to $n_{R}\left(2006\right)=27.5\%$ yields:

$\Delta R_{0}=1-\frac{R_{0}\left(2006\right)}{R_{0}\left(2005\right)}=1-\frac{R_{0}\left(0\right)\left(1-n_{R}\left(2006\right)\right)}{R_{0}\left(0\right)\left(1-n_{R}\left(2005\right)\right)}=12.2\%$.

### 3. Trend analysis of the prevalence in ARQ*/VRQ animals in 2005-2008

From the active surveillance data and genotyping samples we obtain the following information for each year *t* (with $t\in${2005, 2006, 2007, 2008}):

- Number of positive animals, $y_{t}$, out of a number of tested animals, $n_{t}$.
- Number of ARQ*/VRQ animals, $x_{t}$, within the $y_{t}$positive animals.
- Number of ARQ*/VRQ animals, $z_{t}$, in a genotyping sample (of negatively tested animals) of size $m_{t}$.

For all four years the numbers$n_{t}$, $y_{t}$, and$m_{t}$are all non-zero. We can therefore define the binomial probabilities$q_{t}$,$Q_{t}$, and $\pi_{t}$by assuming:

$$y_{t}|n_{t}\sim Bin\left(n_{t},q_{t}\right)$$

$$x_{t}|y_{t}\sim Bin\left(y_{t},Q_{t}\right)$$

$$z_{t}|m_{t}\sim Bin\left(m_{t},\pi_{t}\right)$$

The model likelihood follows as a product of binomial probabilities. The maximum-likelihood estimators of these probabilities are: . The underlying probability$p_{t}$of scrapie in ARQ*/VRQ animals can be expressed in terms of the binomial probabilities as follows:

$$p_{t}=P_{t}(\text{animal found positive}|\text{genotype of animal is ARQ*/VRQ})=\frac{q_{t}Q_{t}}{q_{t}Q_{t}+(1-q_{t})\pi_{t}}.$$

The maximum-likelihood estimator is given by:

(A.2)

We reparameterize the model in terms of $q_{t}$, $Q_{t}$, and $p_{t}$ by rewriting:

$z_{t}|m_{t}\sim Bin\left(m_{t},\frac{\left(1-p_{t}\right)q{}_{t}Q_{t}}{p_{t}\left(1-q_{t}\right)}\right)$ .

The trend analysis is performed by assuming the model:

$logit(p_{t})=a+b\tilde{t}$,

where $\tilde{t}\equiv t-2005,$ and by testing the null hypothesis $b=0$ using the likelihood-ratio test (maximizing the likelihoods numerically). In 2005, apart from the 27 scrapie cases out of $y_{2005}=35$ that were found to be of ARQ*/VRQ genotype, there were two scrapie cases of unknown genotype. For this trend analysis we conservatively assume $x_{2005}=27$.

**References**

[1] Anderson RM, May RM, Infectious diseases of humans: dynamics and control, Oxford and New York, Oxford University Press, 1991.

[2] Keeling MJ, Rohani P, Modeling Infectious Diseases in Humans and Animals, Princeton, Princeton University Press, 2008.
